# Germline mosaicism in X-linked periventricular nodular heterotopia

**DOI:** 10.1186/1471-2377-14-125

**Published:** 2014-06-07

**Authors:** Monique M LaPointe, Elizabeth L Spriggs, Aizeddin A Mhanni

**Affiliations:** 1 Department of Biochemistry and Medical Genetics Faculty of Medicine, University of Manitoba, Winnipeg, MB, Canada; 2 Department of Pediatrics and Child Health, University of Manitoba, Winnipeg, MB, Canada; 3 Genetics and Metabolism Program, FE229-820 Sherbrook Street, Winnipeg, MB R3A 1R9, Canada

**Keywords:** X-linked periventricular nodular heterotopia, Germline mosaicism, Genetic counseling

## Abstract

**Background:**

X-linked periventricular nodular heterotopia is a disorder of neuronal migration resulting from mutations in the filamin A gene. This is an X-linked dominant condition where most affected patients are female and present with seizures. Extra–cerebral features such as cardiac abnormalities and thrombocytopenia have also been documented. Loss of function mutations in filamin A are predicted to result in prenatal lethality in males. Somatic mosaicism and mutations that lead to partial loss of function of the protein are hypothesized to explain viability of males reported in the literature. We report the first case of germline mosaicism involving a loss of function mutation in filamin A in a family where brain MRI, clinical exam, and mutation analysis is normal in both biological parents.

**Case presentation:**

The index patient, a 39 year old female with normal development, had her first seizure at 24 years with no evidence of any precipitating factors. Brain MRI shows bilateral periventricular nodular heterotopia. She has thrombocytopenia and an echocardiogram at age 32 years revealed a mildly dilated aortic root and ascending aorta with mild aortic regurgitation. The second patient, the 36 year old younger sister of the index case, is currently healthy with no evidence of seizures or cardiac abnormalities. Her brain MRI is consistent with bilateral periventricular nodular heterotopia. The mother is healthy at 57 years of age with a normal brain MRI. The father is healthy at 59 years of age with a normal brain MRI. DNA sequencing of lymphocyte extracted DNA from the two sisters shows a c.2002C > T transition in exon 13 of filamin A resulting in a p.Gln668Ter mutation. This nonsense mutation was not detected in peripheral blood lymphocytes from the unaffected parents.

**Conclusion:**

This report provides evidence for germline mosaicism in filamin A-associated periventricular nodular heterotopia. This case must now be considered when providing genetic counseling to families where a proband presents as an isolated case and parental investigations are unremarkable.

## Background

Periventricular nodular heterotopia (PNH) is a disorder of cortical development [1]. PNH is a term used to describe the collections of neurons lining the lateral ventricles that have failed to migrate normally to form the cerebral cortex [1]. It is a clinically and genetically heterogeneous group of disorders [2]. Mutations in the filamin A gene *(FLNA)* result in an X-linked dominant form of this disorder [3]. Mutations in *FLNA* leading to protein truncation are the predominant cause of the PNH phenotype [2,4]. Most affected females present with seizures and normal to mildly impaired cognitive function [2,5]. *FLNA*-associated PNH may also be associated with other cerebral malformations as well as extra-cerebral features [6]. The condition typically results in prenatal lethality or a more severe phenotype in males although paternal transmission has been documented in the literature [2,7-9].

Mutations in *FLNA* are associated with a wide spectrum of disorders including the otopalatodigital syndrome (OPD) spectrum disorders [10]. The phenotypes of these disorders are clinically distinct from *FLNA*-associated PNH and result from mutations that conserve the reading frame [10]. *FLNA* post-zygotic mutations and germline mosaicism have been reported in the OPD spectrum disorders [10].

We present the first documented evidence for germline mosaicism in a family with two sisters with *FLNA*-associated PNH due to a loss of function mutation in *FLNA* and clinically unaffected parents.

## Case presentation

**Figure 1:**
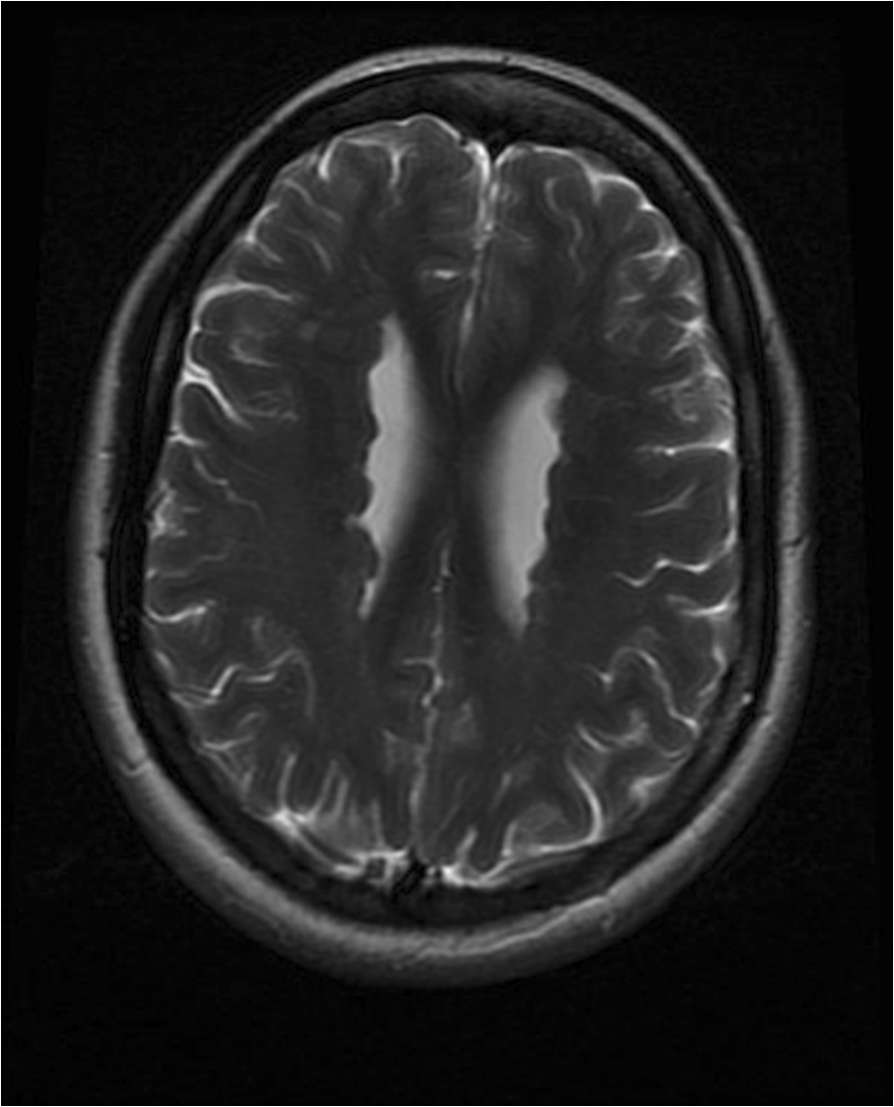
Brain MRI of the index patient showing PNH.

**Figure 2:**
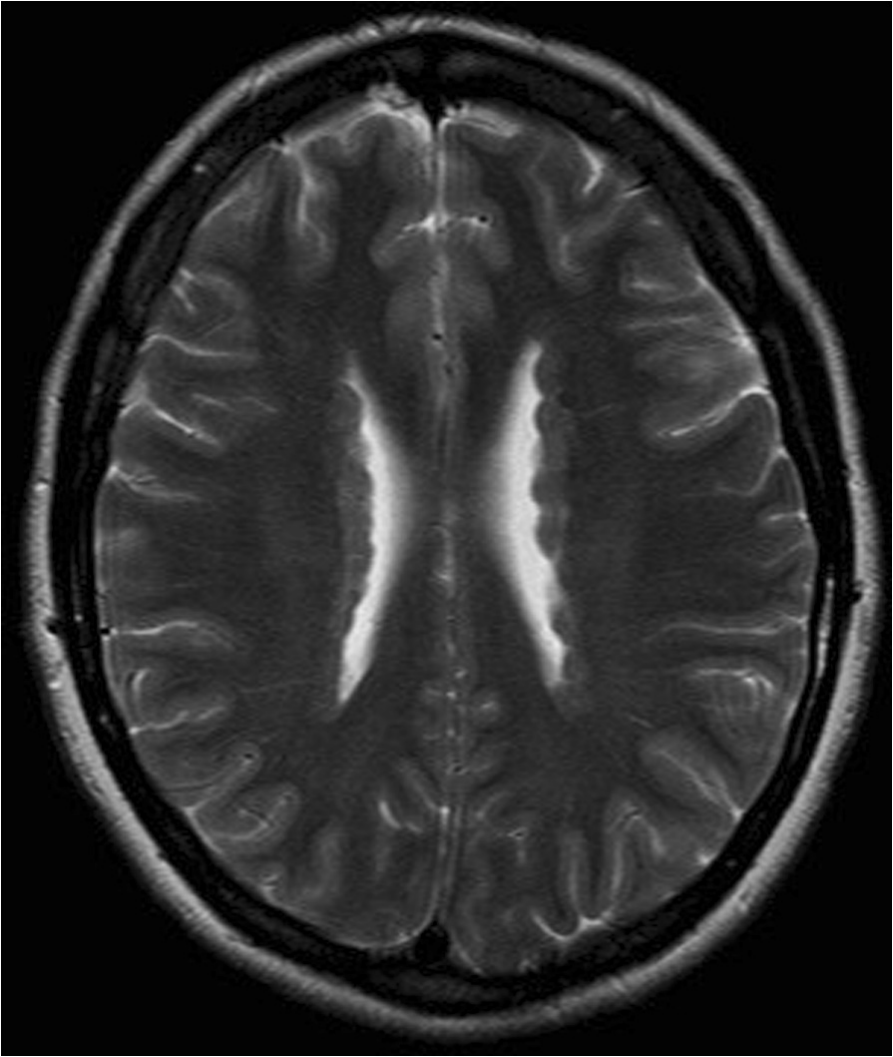
Brain MRI of the sister showing PNH.

The index patient, a 38 year old female, was born after an uneventful pregnancy and delivery. Shortly after delivery she was found to have an ectopic 3rd kidney and a patent ductus arteriosus which required surgical closure at 18 months of age. Her development was normal with no history of seizures, meningitis, encephalitis or major head trauma. She was noted to have thrombocytopenia at age 23 years after complaints of unexplained weight loss. Bone marrow aspiration did not reveal any abnormalities. She continues to have easy bruising and prolonged bleeding time. She had her first seizure at 24 years with no evidence of any precipitating factors. Her neurologic examination was normal. Brain magnetic resonance imaging (MRI) revealed bilateral multiple and diffuse PNH (Figure 1). She has episodic headache with photophobia and phonophobia. An echocardiography at age 32 years revealed a mildly dilated aortic root and ascending aorta with mild aortic regurgitation.The second patient, a younger sister of the index case, is now 36 years of age. Her medical history is relevant for migraine headaches with visual aura. Her neurologic examination was normal and her EEG did not show any interictal abnormalities. An MRI of the brain shows heterotopic gray matter of both hemispheres (Figure 2). Her echocardiogram was normal.

The parents are a healthy nonconsanguineous couple. The mother is now 56 years. She is of English, Irish, and Belgian ethnic background. Her past medical history is unremarkable. Her brain MRI does not show evidence of PNH. The father is now 58 years. He is of Ukrainian and Polish ethnic background. His past medical history is unremarkable. His brain MRI does not show evidence of PNH.

### Mutation analysis

DNA sequencing revealed both sisters to be heterozygous for a c.2002C > T transition in exon 13 of the *FLNA* gene resulting in a p.Gln668Ter mutation. This nonsense mutation has previously been reported as a *FLNA* disease-causing mutation in a patient with PNH [5]. Analysis of lymphocyte extracted DNA from the mother and father did not reveal the *FLNA* exon 13 mutation. Parental relationships were verified molecularly using the Identifier kit (Applied Biosystems).

## Conclusions

We describe two sisters with PNH due to a *FLNA* nonsense mutation. This mutation was not identified in extracted DNA from the lymphocytes of the clinically unaffected parents. Brain MRI in both parents does not show PNH. The most likely explanation for this finding is germline mosaicism.

It is possible that either parent is also somatic mosaic for this *FLNA* mutation as Sanger sequencing may not detect low levels of mosaicism [11]. Furthermore, mutation detection in the parents was limited to peripheral lymphocytes. Somatic mosaicism has been reported in males and females with *FLNA*-PNH. All individuals in these studies had PNH identified on brain MRI although clinically they may have been asymptomatic [8,9,12]. Somatic and germline mosaicism was reported in a male with a deleterious splice site mutation in *FLNA*[9]. In contrast to our family however, this individual had PNH identified on brain MRI and had other clinical features consistent with the *FLNA*-PNH phenotype [9].

In view of this case report, germline mosaicism should be considered in counseling families where there is an isolated case of *FLNA*-associated PNH and neither parent has any clinical, radiographic or molecular evidence of *FLNA*-associated PNH. Despite the apparent low risk of recurrence in such cases, prenatal testing should be considered for subsequent pregnancies.

## Consent

Written informed consent was obtained from the patient for publication of the case report and any accompanying images. A copy of the written consent is available for review by the Editor of this journal.

## Abbreviations

PNH: Periventricular nodular heterotopia; *FLNA*: Filamin A gene.

## Competing interests

The authors declare that they have no competing interests.

## Authors’ contributions

MML and AAM contributed to the clinical encounter and work up of the family. MML, ELS and AAM contributed to the writing of the manuscript. ELS carried out the molecular genetic studies to confirm parentage. All authors read and approved the final manuscript.

## Pre-publication history

The pre-publication history for this paper can be accessed here:

http://www.biomedcentral.com/1471-2377/14/125/prepub
